# Publisher Correction: Solvent-assisted programming of flat polymer sheets into reconfigurable and self-healing 3D structures

**DOI:** 10.1038/s41467-018-04835-z

**Published:** 2018-06-07

**Authors:** Yang Yang, Eugene M. Terentjev, Yen Wei, Yan Ji

**Affiliations:** 1 The Key Laboratory of Bioorganic Phosphorus Chemistry and Chemical Biology (Ministry of Education), Department of Chemistry, Tsinghua University, 100084 Beijing, China; 2 Cavendish Laboratory, University of Cambridge, CB3 0HE Cambridge, UK

Correction to: *Nature Communications* 10.1038/s41467-018-04257-x, published online 15 May 2018

The original version of this Article omitted the Received and Accepted dates; they should have been 21st January 2018 and 12th April 2018, respectively. This has been corrected in the PDF and HTML versions of the Article.

