# Supplementary material for: Solvent-assisted programming of flat polymer sheets into reconfigurable and self-healing 3D structures
Source: Nat Commun. 2018 May 15;9:1906. doi: 10.1038/s41467-018-04257-x (PMC5954017; doi:10.1038/s41467-018-04257-x)
Supplement: Supplementary file 1 — Supplementary Information [file 41467_2018_4257_MOESM1_ESM.pdf]

Supplementary information

Solvent-assisted programming of flat polymer sheets into reconfigurable and self-healing 3D structures

Yang Yang[1, 2], Eugene M. Terentjev[2], Yen Wei[1], Yan Ji[1,*]

[1]The Key Laboratory of Bioorganic Phosphorus Chemistry & Chemical Biology (Ministry of Education), Department of Chemistry, Tsinghua University, Beijing 100084, China.

[2]Cavendish Laboratory, University of Cambridge, Cambridge CB3 0HE, U. K.

Correspondence and requests for materials should be addressed to Y. Ji (email: jiyan@mail.tsinghua.edu.cn)

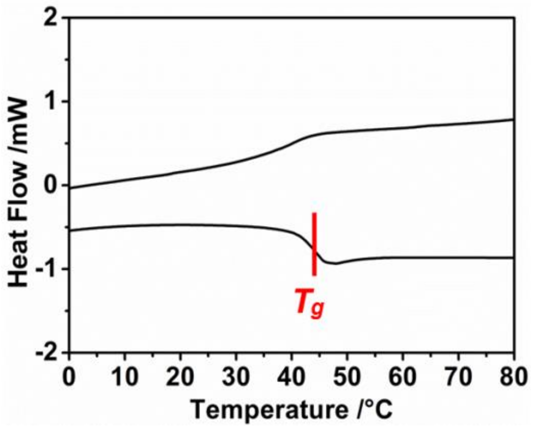

Supplementary Figure 1. DSC traces for both heating/cooling (rate of 10 ℃/min) of vitrimer. The glass transition temperature (T_g) is about 44 ℃.

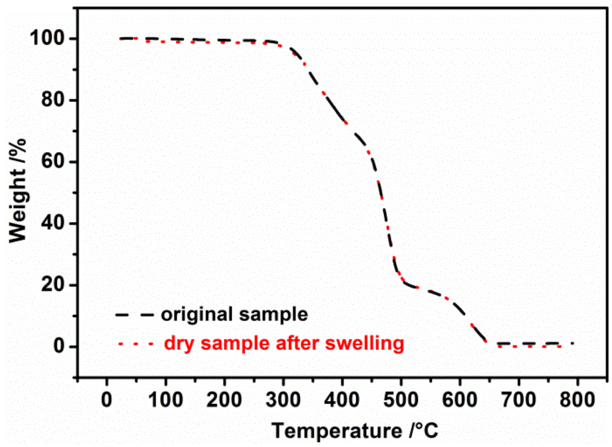

Supplementary Figure 2. TGA curves of original sample and dry sample after swelling, which show that THF in dry sample after swelling has fully evaporated.

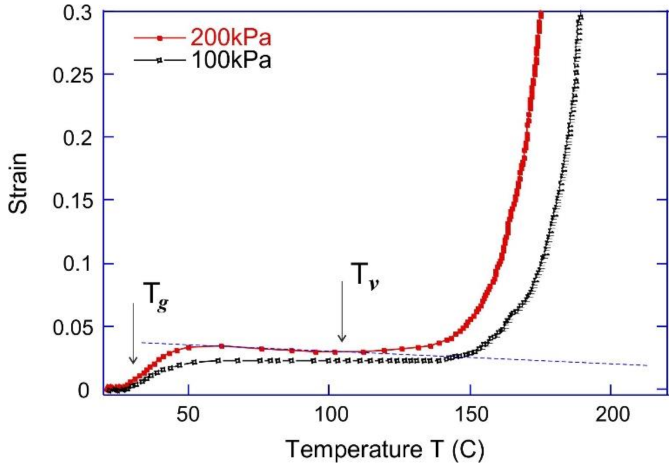

Supplementary Figure 3. Dilatation curves at two different constant loads. The dashed line shows the rubber-elastic regime ($G = n_c k_B T$), leading to a contraction on heating. The arrows mark the glass and the vitrification transitions.

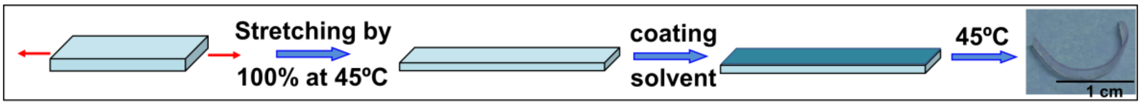

Supplementary Figure 4. A sketch of preparing bending sample.

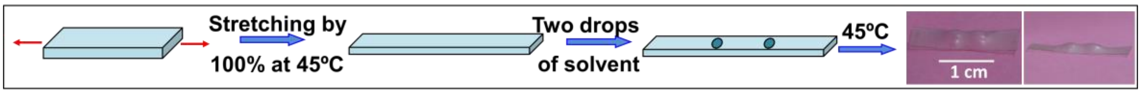

Supplementary Figure 5. A sketch of preparing sample with dot pattern.

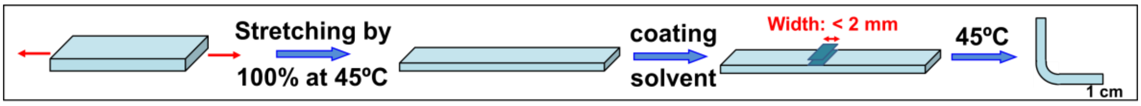

Supplementary Figure 6. A sketch of the first way to prepare folding samples.

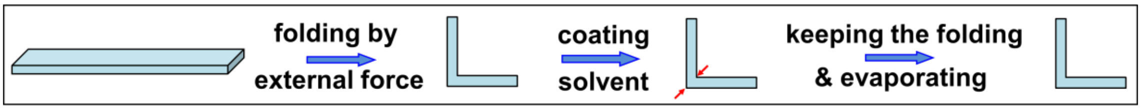

Supplementary Figure 7. A sketch of the second way to prepare folding samples.

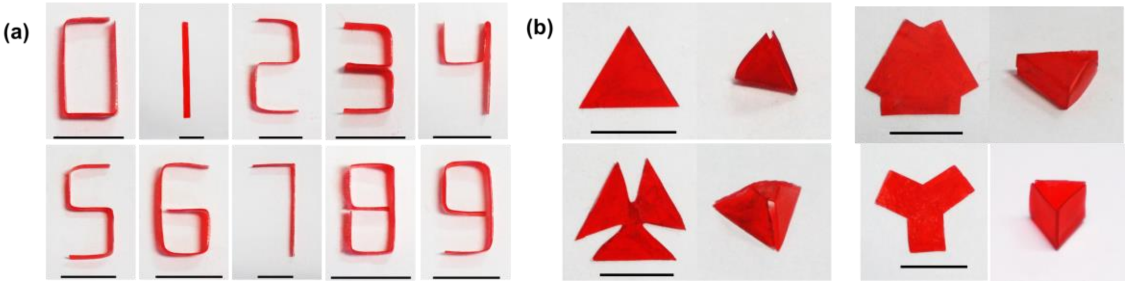

Supplementary Figure 8. The folding samples made by the second way.

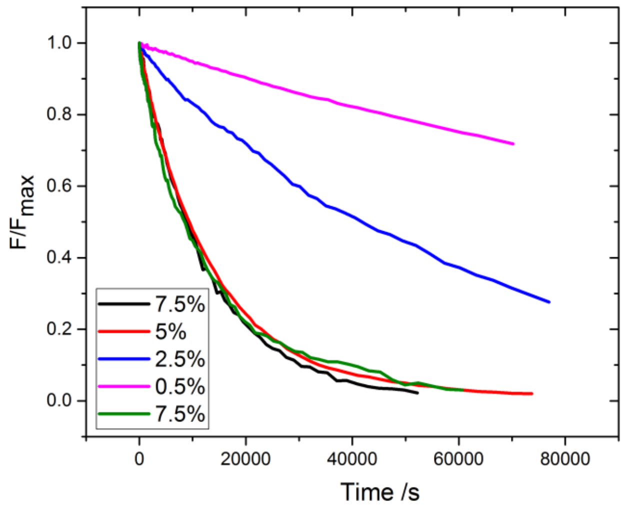

Supplementary Figure 9. Scaled stress-relaxation curves for transesterification vitrimer at T=117 ℃ and several catalyst concentrations (two separately prepared samples with 7.5% TBD are presented for control), as labelled in the plot.

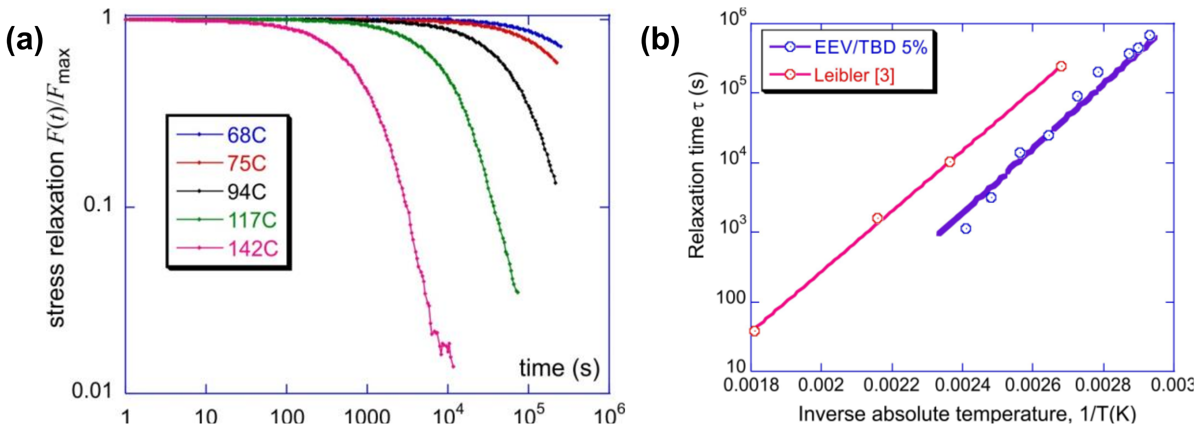

Supplementary Figure 10. (a) Scaled relaxation curves for transesterification with 5 mol% of TBD catalyst, at several temperatures labelled in the plot. The log-log plot allows a better view of fast high-temperature processes, and universal shape of the curves, yet the fitting of these curves is a simple exponential, with good accuracy. (b) The Arrhenius plot for the stress relaxation time. Blue symbols: our data, red symbols: the data from Leibler et al.[2] The fitting gives the bond strength $\Delta G \approx 19$ kcal/mol for transesterification/TBD, and ≈20 kcal/mol for transesterification/ZnAc.

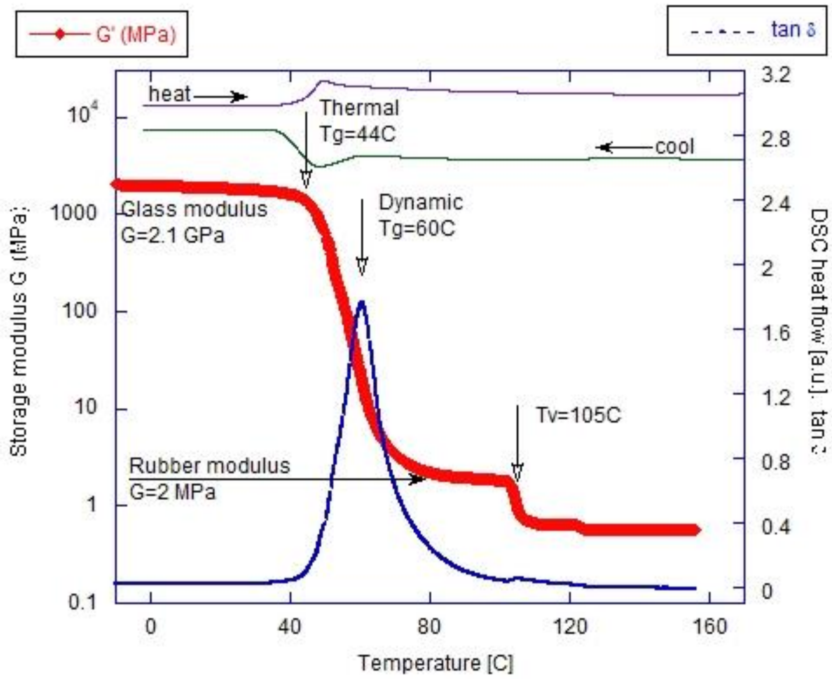

Supplementary Figure 11. The dynamic-mechanical test of the solvent-free vitrimer, varying temperature at a constant (low) frequency of 1 Hz. The storage modulus *G'* and the loss factor tan δ indicate the dynamical changes that occur at the glass transition, and at the elastic-plastic transition. The plot also shows the DSC data obtained at heating/cooling rate of 3 ℃ /min.

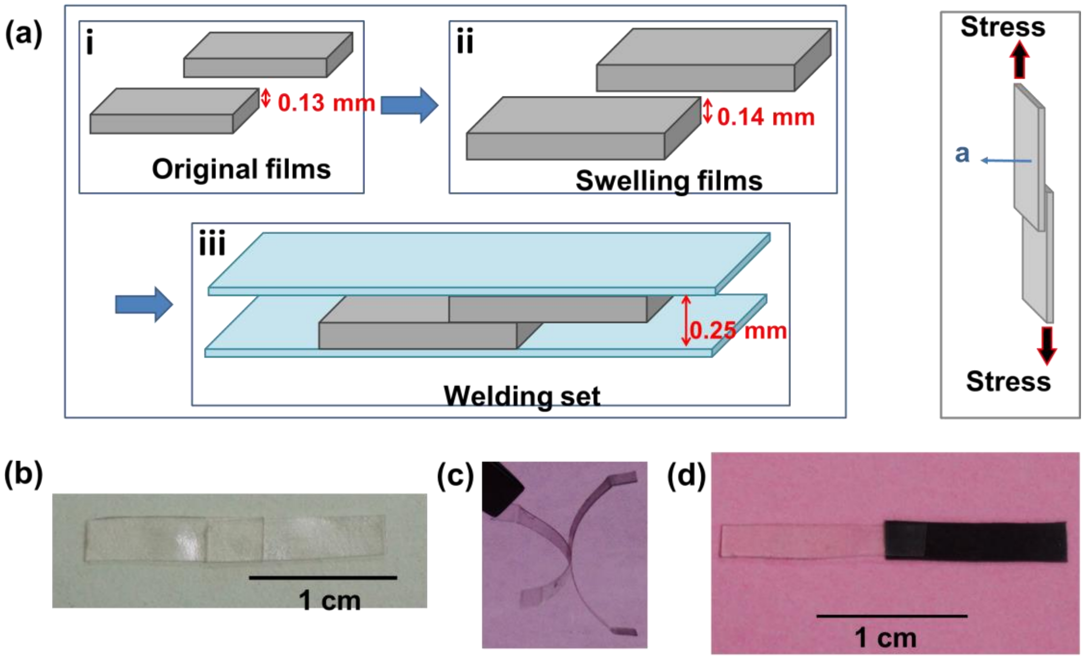

Supplementary Figure 12. (a) The Illustration of welding set. (i) before swelling, (ii) swelling sample, (iii) the welding set. The right pannel is an illustration of how lap shear test operated by DMA. (b) Illustration of welding the overlap areas by solvent. (c) Welding to more complicated structures. (d) Welding epoxy vitrimer with other component by solvent.

Supplementary Note 1. Characterization of the vitrification temperature

To determine the vitrification temperature, or the point at which the elastic-plastic transition occurs in the vitrimer, we need to follow the procedure of Pritchard et al.[1], by identifying a point at which the rubber-elastic (entropic) modulus $G = n_c k_B T$ first starts to diminish (deviate from the linear T-dependence). On a typical 'dilatation curve' monitoring the uniaxial extension under constant load, on increasing temperature, the rubber-elastic regime shows by the linear diminishing slope (as the modulus G grows on heating, the strain decreases). The vitrification temperature is defined as the point of first deviation from this regime (labelled on the plot in Supplementary Figure 3); in the broad range of loads T_v remains approximately invariant, $T_v \approx 105$ ℃.

Supplementary Note 2. The correlation between the bending curvature and the pre-stretched ratio

In Supplementary Figure 4, the bending curvature increases as the pre-stretched ratio increases from 10% to 100%, but the sample is easy to break when adding solvent, if it is pre-stretched too much. So we pre-stretched only up to 100% in our tests.

Supplementary Note 3. The correlation between the amount of solvent and the folding angle

The amount of solvent impacts folding angle in Supplementary Figure 6. The folding angle caused by coating two-layer THF is smaller than that caused by coating one-layer THF. And if we coated three-layer THF, the bottom layer is swelled as well. So we coated two-layer THF when preparing folding sample, whose dose of THF is about double-dose used to prepare bending sample. For example, when the size of sample is 2 cm x 2.5 mm x 0.2 mm, we coat 40 µL to the area of 2 mm x 2.5 mm to prepare a folding sample; while when preparing a bending sample, the same area size (2 mm x 2.5 mm) is coated 20 µL.

Supplementary Note 4. Influence of TBD contents on stress relaxation

Clearly, the role of TBD is crucial in transesterification. To test it in more detail, we compared the stress relaxation dynamics at different loading of TBD. Supplementary Figure 9 shows the relaxation curves, which fit to a simple exponential with only one free parameter: relaxation time $\tau(T)$. It is clear from the plotted data that the transesterification becomes faster as catalyst loading increases, but this effect saturates at TBD concentration above 5 mol%. We suppose that this is because the TBD is fully bonded into the network, and further addition of it has no additional effect. The strong basicity of the guanidine group in TBD speeds up the transesterification when the guanidine group increases.

Supplementary Note 5. Influence of temperature on stress relaxation

Temperature also affects stress relaxation greatly. Supplementary Figure 10a shows results of a typical stress-relaxation at a series of temperatures. It is presented via a scaled relaxation function $F(t)/F_{max}$, in order to focus purely on the time dependence. Since the time of relaxation is very different at different temperatures, we follow the example of earlier papers and plot it in log-log scale. Importantly, and highly unusually in polymer networks, these

relaxation curves are very well fitted by a simple exponential function: $\exp[-t/\tau]$, with only a single fitting parameter: the relaxation time $\tau(T)$, the inverse of the rate β of the earlier discussion. Supplementary Figure 10b shows the Arrhenius plot of our relaxation experiments on networks, that is plotting $\ln[\tau] = \mathrm{const} + \Delta G/k_B T$, with temperature in absolute (Kelvin) units. For comparison, we also digitized the data from the original paper of Leibler et al. who used a different catalyst.[2, 3] It is clear that both data sets show a very clean single-value of activation energy. In our tests, we deliberately probed much lower temperatures (far below the vitrification point T_v) where the transesterification is slow. We obtained the bond strength $\Delta G \approx 18.9$ kcal/mol (which corresponds to about 33 $k_B T$ at room temperature) with 5 mol% of TBD catalyst. In Leibler's case, the zinc acetate catalyst was used, also at 5 mol%, and our fitting gives $\Delta G \approx 19.8$ kcal/mol (or 34 $k_B T$). So, combining with the infomation in Fig. S7, it is clear that the presence catalyst (TBD in our case) is the key to the transesterification at high temperature.

Supplementary Note 6. Calculation of tension forces in swollen vitrimer network

Here we present the details of the calculation that leads us to understand the mechanism of solvent-induced transesterification, and the resulting elastic-plastic transition of the vitrimer. First of all the dynamic-mechanical characterization of the dry vitrimer sample, Supplementary Figure 11, gives the onset of the glass transition at ~44 ℃ (in agreement with calorimetric data in Supplementary Figure 1), and the true dynamic-mechanical transition (identified by the peak in the loss factor $\tan\delta$) at T_g = 60 ℃. The elastic-plastic transition occurs at the vitrification temperature T_v = 105 ℃. At this point it is important to remind the reader that there have been different versions of "vitrification temperature" used in the literature. The original work of Leibler[2], as well as our own, suggested that 'vitrification' occurs at a relatively high temperature: when the fast plastic flow occurs under stress. However, a careful study of Pritchard et al.[1] has shown that the true elastic-plastic transition sets in at a much lower temperature, identified by the first deviation of the constant-stress modulus from the rubber-elastic value (which is linearly proportional to the absolute temperature). That transition occurs at 105 ℃ in our material, and the data plotted in Supplementary Figure 11 shows this clearly, confirming the result of a thermal dilatation test in Supplementary Figure 3.

Given the rubber modulus of the dry network (just below T_v) is $G \approx 2$MPa, the basic theory of rubber elasticity ($G = n_c k_B T$) gives us the estimate value of crosslinking density, so that the average equilibrium distance between crosslinks (the mesh size, or the span of the polymer network) is $R = n_c^{-1/3} \approx 1.4$nm. The equilibrium swelling factor of this network was obtained by measuring the volume increase after several days of swelling in THF, giving $Q \approx 1.75$ (and a similar value in DCM). This gives two relevant values: the relative increase of linear dimensions (strain): $\lambda = Q^{1/3} \approx 1.21$ (i.e. 21% increase in the mesh size R), and the approximate number of monomers on a network strand between crosslinks: $N = Q^{8/3} \approx 4.5$ (this is a textbook[4] estimate under the assumption of ideal Gaussian chains).

One may reasonably question whether the textbook ideal-chain equations are applicable for polymer strands with only 4-5 monomers. However, it is not uncommon in practice that such short chains produce correct entropically-driven expressions in rubber elasticity. The other factor to be concerned about is why does the assumption that equilibrium swelling ratio Q not depend on the chemical miscibility (e.g. in the form of Flory χ-parameter). It is easy to show that for the equilibrium solvent concentration in the swollen gel[4]: $\varphi = (Q-1)/Q$, there is

indeed a very weak dependence on χ when χ<<1, which is the practical condition for a 'good solvent'.

Given the parameters of vitrimer network determined above, and the monomer size $b \approx 0.5$nm, we can now estimate the tension force acting on a crosslink from each of the four strands connected to it. Remaining in the simplest approximation of ideal Gaussian chains, this tension force is: $F = \left(\frac{3k_BT}{b^2N}\right) R \approx 19$ pN at room temperature. Swelling will increase this force by about 20% due to the increase of the mesh size R. Since each strand elongates by $(\lambda - 1)R \approx 0.3$nm, the mechanical work required to stretch the four strands under tension can be estimated as:

$$\Delta W = 4 \cdot \int_R^{\lambda R} \left(\frac{3k_BT}{b^2N}\right) x \cdot dx \approx 2.7 \cdot 10^{-20}\,\text{J} = 3.9\ \text{kcal/mol}$$

This shifts the value of the activation energy for the transesterification reaction, such that its reaction rate will now be given by:

$$k_{BER} = \omega_0 \cdot e^{-\frac{\Delta G - \Delta W}{k_BT}}$$

So if the 'dry' vitrification temperature (when ΔW=0) was T_v = 105 ℃, then the same reaction rate will be achieved in the stretched (swollen) network at $T_{sw} \approx$ 25 ℃.

Supplementary Note 7. Lap-shear test of the welded sample

We used lap shear test to show the adhesion of the welded sample here. Lap shear test is now a normally used method to characterize the adhesion of welded sample. The Supplementary Figure 12a (right pannel) is an illustration of how lap shear test operated by DMA. If the sample is well welded, when stretched, it breaks at the bulk materials (such as the point a) instead of sliding form the overlapped part. This lap shear test is a bit different from peel test, but results of both methods are coincident. Here the welded sample breaks in the regions of bulk materials instead of sliding form the overlapped part, which indicates a strong joint.

Supplementary References

1. Pritchard, R. H., Redmann, A.-L., Pei, Z., Ji, Y. & Terentjev, E. M. Vitrification and plastic flow in transient elastomer networks. *Polymer* **95**, 45-51 (2016).
2. Montarnal, D., Capelot, M., Tournilhac, F. & Leibler, L. Silica-like malleable materials from permanent organic networks. *Science* **334**, 965-968 (2011).
3. Meng, F., Pritchard, R. H. & Terentjev, E. M. Stress relaxation, dynamics, and plasticity of transient polymer networks. *Macromolecules* **49**, 2843-2852 (2016).
4. Rubinstein, M. & Colby, R. H. *Polymer physics*. Oxford University Press (2003).
